# Effect of sigh in lateral position on postoperative atelectasis in adults assessed by lung ultrasound: a randomized, controlled trial

**DOI:** 10.1186/s12871-022-01748-9

**Published:** 2022-07-11

**Authors:** Caifeng Li, Qian Ren, Xin Li, Hongqiu Han, Min Peng, Keliang Xie, Zhiqiang Wang, Guolin Wang

**Affiliations:** 1 Department of Critical Care Medicine, Tianjin Medical University General Hospital, NO.154, Anshan Road, Heping District, Tianjin, China; 2 Advertising Center, Tianjin Daily, Tianjin, China; 3 Department of Cardiothoracic Surgery, Tianjin Medical University General Hospital, Tianjin, China; 4 Department of General Surgery, Tianjin Medical University General Hospital, Tianjin, China

**Keywords:** Sigh, Lateral position, Recruitment maneuver, Atelectasis, General anesthesial, Lung ultrasound

## Abstract

**Background:**

Postoperative atelectasis occurs in 90% of patients receiving general anesthesia. Recruitment maneuvers (RMs) are not always effective and frequently associated with barotrauma and hemodynamic instability. It is reported that many natural physiological behaviors interrupted under general anesthesia could prevent atelectasis and restore lung aeration. This study aimed to find out whether a combined physiological recruitment maneuver (CPRM), sigh in lateral position, could reduce postoperative atelectasis using lung ultrasound (LUS).

**Methods:**

We conducted a prospective, randomized, controlled trial in adults with open abdominal surgery under general anesthesia lasting for 2 h or longer. Subjects were randomly allocated to either control group (C-group) or CPRM-group and received volume-controlled ventilation with the same ventilator settings. Patients in CPRM group was ventilated in sequential lateral position, with the addition of periodic sighs to recruit the lung. LUS scores, dynamic compliance (Cdyn), the partial pressure of arterial oxygen (PaO_2_) and fraction of inspired oxygen (FiO_2_) ratio (PaO_2_/FiO_2_), and other explanatory variables were acquired from each patient before and after recruitment.

**Results:**

Seventy patients were included in the analysis. Before recruitment, there was no significant difference in LUS scores, Cdyn and PaO_2_/FiO_2_ between CPRM-group and C-group. After recruitment, LUS scores in CPRM-group decreased significantly compared with C-group (6.00 [5.00, 7.00] vs. 8.00 [7.00, 9.00], *p* = 4.463e-11 < 0.05), while PaO_2_/FiO_2_ and Cdyn in CPRM-group increased significantly compared with C-group respectively (377.92 (93.73) vs. 309.19 (92.98), *p* = 0.008 < 0.05, and 52.00 [47.00, 60.00] vs. 47.70 [41.00, 59.50], *p* = 6.325e-07 < 0.05). No hemodynamic instability, detectable barotrauma or position-related complications were encountered.

**Conclusions:**

Sigh in lateral position can effectively reduce postoperative atelectasis even without causing severe side effects. Further large-scale studies are necessary to evaluate it’s long-term effects on pulmonary complications and hospital length of stay.

**Trial registration:**

ChiCTR1900024379. Registered 8 July 2019,

**Supplementary Information:**

The online version contains supplementary material available at 10.1186/s12871-022-01748-9.

## Introduction

For decades, surgeons have noticed that many patients with previously normal lung function would suffer from oxygenation compromise after surgery under general anesthesia [1], but the underlying mechanism is still unclear. It was not until 1963 that Bendixen first proposed that progressive lung collapse or atelectasis might be the principal cause of postoperative hypoxemia [2]. However, due to the limitation of imaging technology, it was difficult to validate this hypothesis in vivo. With the emergence of advanced imaging methods, such as computed tomography (CT), magnetic resonance imaging (MRI), electric impedance tomography (EIT) and ultrasonography [3, 4], atelectasis was reported in 90% of patients undergoing general anesthesia [1, 4, 5]. Another study showed that atelectasis even occurred in 100% of patients on CT scan after general anesthesia [6]. Mild atelectasis might recover spontaneously with no lasting harm, while severe atelectasis could impair gas exchange, decrease lung compliance [7], and even cause other serious complications, including pneumonia, acute respiratory failure, weaning failure or reintubation within 48 h [8], which are independently associated with a longer hospital stay, higher in-hospital mortality rate [9, 10], and greater hospital cost [11].

Given such undesirable side effects of atelectasis, recruitment maneuvers have been proposed to reopen collapsed lung units and improve blood oxygenation by intentional transient increase in alveolar pressure. However, studies have shown that up to 50% of patients did not obtain significant improvement in gas exchange from recruitment maneuvers [12, 13]. Reason for the inconsistent results of recruitment maneuvers is the inhomogeneous distribution of atelectasis, which results in a preferential inflation of the already opened lung zones rather than the targeted collapsed lung areas [14]. Moreover, recruitment maneuvers could significantly increase intrathoracic pressure, decrease left ventricular end-diastolic volume as well as cardiac output [15]. Although other serious adverse events, such as barotrauma and arrhythmia, are uncommon [16], it would be a disaster for critical patients once they occur.

It has been reported that many natural behaviors, such as crying, coughing, sneezing, sighing or postural changes, have recruiting effects [17]. Among healthy people, periodic sighs and intermittent adjustments of body position counter local tendency for collapse and prevent progression of atelectasis [18–22]. However, most of these physiological recruitment maneuvers, executed spontaneously and subconsciously, would be interrupted by general anesthesia. That is why atelectasis occurs quickly under general anesthesia [23]. While continued postoperative analgesia and sedation would inhibit the recovery of these self-protection behaviors and aggravates atelectasis [24]. Therefore, we reasoned that if we could simulate these natural behaviors after surgery, we might ameliorate or reverse postoperative atelectasis actively.

To maximize the benefits and minimize the complications of recruitment maneuvers, adequate monitoring is necessary. Lung mechanics and intermittent blood gas analysis, or pulse oximetry and end-tidal CO_2_ pressure, have been reported to be the most commonly used parameters to guide ventilator setting and assess lung recruitment, but they cannot monitor lung aeration directly and dynamically [25]. In recent years, LUS has earned a special place among all imaging techniques integrating both clinical and instrumental assessment of critical patients. B-lines, an ultrasound artifact representing extravascular lung water [26], are similarly correlated significantly with loss of peripheral lung aeration [27] and anesthesia-induced atelectasis [28]. Therefore, LUS can be used to monitor lung aeration in a real-time fashion during recruitment maneuvers [29–32].

So, we conceived a randomized controlled trial (RCT) to verify the clinical effectiveness and feasibility of a combined physiological recruitment maneuver (sigh in lateral position) by using LUS, which can be defined as an active reopening of atelectatic lung tissue by simulating intermittent sigh breaths and intentional postural changes rather than by elevating airway pressure.

## Patients and methods

### Study design

This study was a prospective, single-center, randomized, controlled trial conducted at a tertiary teaching hospital in the Republic of China between August 2019 and December 2019. The study was approved by Tianjin Medical University General Hospital Institutional Review Board and was registered before patient enrollment at www.chictr.org.cn with a Clinical Trial Number as ChiCTR1900024379. This study was carried out in accordance with the 2013 Declaration of Helsinki and its later amendments. Informed consent was obtained from all participants.

### Inclusion and exclusion criteria

Patients were screened by an investigator (KLX) for eligibility if they fulfilled the predefined inclusion criteria: age more than 18 years old; scheduled for elective open abdominal surgery under general anesthesia with endotracheal intubation lasting for 2 h or longer. Patients were excluded from the study if they were pregnant women; had pre-existing lung diseases with abnormal CT scans; had previous thoracic procedures (such as thoracotomy or video-assisted thoracoscopy); had perioperative pneumothorax or subcutaneous emphysema; had contraindications for lateral position (such as unstable spinal fractures).

### Randomization and masking

The enrolled patients were randomly assigned to either C-group or CPRM-group by an investigator (MP) using a random number generator (www.random.org) with an allocation ratio of 1:1. The randomization assignment and group allocation information were concealed by the same investigator (MP) in opaque, sealed, sequentially numbered envelopes containing the guidance for the attending intensivist. The operator of LUS (CFL), the reviewer of ultrasound images (CFL and XL) and the statistical analyst (HQH) were blinded to the patients’ group allocation.

### Analgesia and sedation protocol

Patients were deeply sedated (Richmond Agitation Sedation Scale score of − 4) with continuous infusion of propofol and fentanyl to avoid confounding influences caused by patients’ spontaneous breathing effort. Peak airway pressure (Ppeak) and tidal volume (Vt) were recorded by a well-trained intensivist (ZQW) during periods of passive breathing, usually by ensuring the observed respiratory rate matched the ventilator setting rate. All patients had an indwelling peripheral arterial catheter for continuous blood pressure monitoring and serial arterial blood gas analysis.

### Recruitment maneuver protocol

Patients were ventilated with volume control mode. The ventilator settings were specified as follows: Vt, 8 ml/kg ideal body weight; respiratory rate (RR), 15 breaths/min; inspiratory-to-expiratory ratio (I/E), 1: 2; positive end-expiratory pressure (PEEP), 5 cm H2O; FiO_2_, 40%-50%. The respiratory rate and oxygen concentration were readjusted accordingly to maintain the partial pressure of arterial carbon dioxide (PaCO_2_) between 35 and 45 mm Hg and keep oxygen saturation above 95%. A combined lung recruitment maneuver, sigh in lateral position, was performed in CPRM-group. This maneuver consisted to move the patient sequentially in: (1) the right lateral position to reopen atelectic zone of the upper left lung (One hour); (2) the left lateral position to recruit atelectic region of the upper right lung (One hour); and (3) finally back to supine position, while periodical sigh breaths was provided by the ventilator automatically every 100 breaths with 2 times of setting tidal volume, keeping other ventilator settings constant with C-group. To maintain lateral position, a pillow was placed in front of the patient’s chest for him/her to embrace and special care was taken to prevent accidental extubation. The entire procedure took approximately 2 h to be completed.

If the lateral position could not be sustained due to agitation or discomfort, patients were returned to the supine position. A dramatic reduction in systolic arterial pressure less than 90 mm Hg or mean arterial pressure less than 60 mm Hg was defined as a serious complication and was unacceptable, intensivists were allowed to stop the recruitment maneuver or change the ventilation protocol at any time for security reasons.

### LUS

LUS was performed by an experienced lung ultrasonographer (CFL), who participated in training programs sponsored by the Chinese Critical Ultrasound Study Group, using a Sonosite M-Turbo portable device (Sonosite Inc., Bothell, WA, USA) with a 1 to 5 MHz convex probe. The settings of the ultrasound machine were as follows: gain, “AUTO GAIN”; penetrability, “GEN”; with “THI” and “MB” both turned off.

**Fig. 1:**
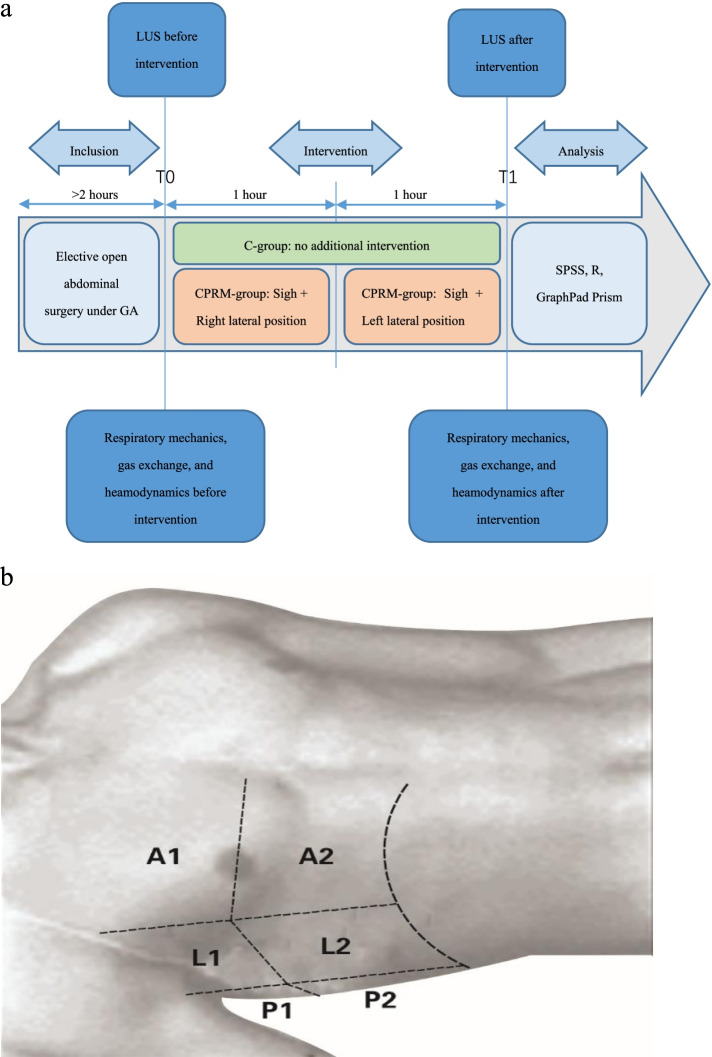
The study protocol. **a** LUS exams were performed for each patient before and after recruitment. T0, before recruitment; T1, after recruitment; C-group, control group; CPRM-group, combined physiological recruitment maneuver group; GA, general anesthesia; LUS, lung ultrasound. **b** Lung areas for LUS. A1, anterosuperior; A2, anteroinferior; L1, laterosuperior; L2 lateroinferior; P1, posterosuperior; P2, posteroinferior; LUS, lung ultrasound

Each hemithorax was divided into anterior, lateral, and posterior lung zones by the anterior and posterior axillary lines, each zone was further separated into upper and lower portions. Therefore, a total of 12 quadrants need to be investigated for both sides. During ultrasound scanning, the probe was positioned longitudinally over intercostal space to obtain a typical image of the bat sign (pleural line) and then placed horizontally along the intercostal space to assess the lung carefully for the following signs: the lung sliding (yielding seashore sign); A-lines (horizontal artifact); B-lines (vertical artifact); juxta-pleural consolidation and/or air-bronchogram. These 12 lung regions were examined sequentially from right to left, cranial to caudal, and anterior to posterior. The anterolateral zones were examined with patients situated in supine position, whereas posterior zones were examined with patients slightly rotated into a lateral position with the help of nurses. In both groups, LUS was carried out at two different time points: before recruitment (T0) and after recruitment (T1). The study protocol was presented in Fig**. **1. All images were saved and exported to a USB disk for subsequent review and further analysis.

### Primary outcome

According to a simple grading system (Supplementary Table S1), LUS score, ranging from 0 to 36, was obtained by summing up the score of 12 individual quadrants to quantitatively assess lung aeration loss [28]. The higher the LUS score, the more severe the aeration loss. All images were analyzed independently by two investigators (CFL and XL) who were blinded to the group allocation; a third researcher (GLW) was invited to resolve any discrepancies between the two researchers.

### Secondary outcome

Vt, Ppeak, PaO_2_ and FiO_2_ were recorded. Variables related to respiratory mechanics and oxygenation were calculated according to the following formulas: Cdyn = Vt/(Ppeak-PEEP); Cstat = Vt/(Pplat-PEEP); Oxygenation index (OI) = PaO_2_/FiO_2_.

### Explanatory analyses

Heart rate (HR), mean arterial pressure (MAP), central venous pressure (CVP), duration of general anesthesia and length of mechanical ventilation were also collected.

### Sample-size calculation

Based on our own clinical experience with LUS score, we considered a mean change of 2 points to be clinically relevant and significant. We hypothesised that a mean difference of 2 points would be observed between C-group and CPRM-group at time point T1. Sample size was calculated by simulating repeated-measure 1-way analysis of variance (ANOVA) using an AR (1) correlation matrix. Correlation value was tried for the covariance matrix. Given an estimated standard deviation (SD) of 3 [33], we calculated that 35 patients in each group should be enrolled with an alpha error of 0.05, a power of 85% and allowing for a dropout rate of 15%.

### Statistical analysis

Continuous variables were tested for normal distribution employing the Shapiro–Wilk method and presented as either the mean (standard deviation, SD) or the median [interquartile range, IQR]. Categorical data were described as numbers and percentages (%). Univariate comparisons between two groups were performed using the Student’s t-test, Mann–Whitney U-test or Analysis of covariance (ANCOVA) with baseline measures as covariates for continuous variables, and the Chi-square test or Fisher’s test for categorical variables. Within-group comparisons (change between T0 and T1) were made employing the Paired t-test or the Wilcoxon signed-rank test as appropriate. A *p-*value less than 0.05 was considered statistically significant. All statistical analyzes were performed using SPSS statistical software, version 24.0 (IBM Corporation, Armonk, NY, USA), R software, version 3.6.1 (Foundation for Statistical Computing, Vienna, Austria) and Graphpad Prism software, version 7 (GraphPad Prism Software, San Diego, California, USA).

## Result

### Characteristics of patients

**Fig. 2:**
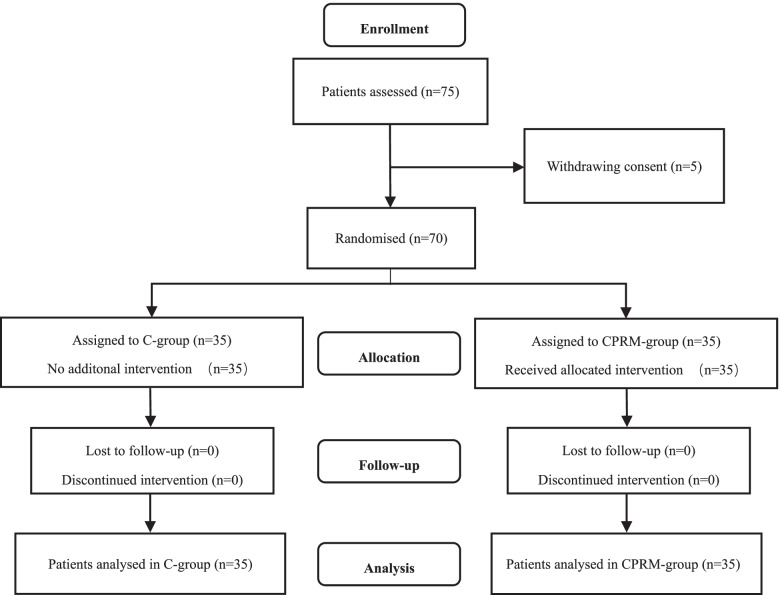
The flowchart of patient selection. C-group, control group; CPRM-group, combined physiological recruitment maneuver group

Between August 2019 and December 2019, Seventy-five consecutive patients for elective abdominal surgery under general anesthesia lasting for 2 h or longer were screened for inclusion. Five patients were excluded due to withdrawing consent (*n* = 5), and thus resulting in 70 evaluable patients included in this study. The flowchart of patient selection is shown in Fig. 2. The general characteristics of the study population are reported in Table 1.

**Table 1:** Clinical characteristics for all patients

	C-group *n* = 35	CPRM-group *n* = 35	*p* value
Age (years)	65.00 [50.50, 69.50]	65.00 [56.00, 72.50]	0.537
Gender, n (%)			0.227
Male	18 (51.4)	12 (34.3)	
Female	17 (48.6)	23 (65.7)	
Height (cm)	166.74 (8.22)	162.89 (7.29)	0.042
Weight (kg)	64.59 (12.27)	61.29 (12.98)	0.278
BMI (kg/m^2^)	23.18 (3.84)	22.98 (4.13)	0.834
ASA physical health status, n (%)			0.445
1–2	25 (71.43)	22 (65.71)	
3–4	10 (28.57)	13 (34.29)	
Comorbidities, n (%)			
Diabetes mellitus	6 (17.14)	7 (20)	0.759
Cardiac disease	5 (14.29)	6 (17.14)	0.743
Cancer	30 (85.71)	26 (74.29)	0.232
Type of surgery, n (%)			0.426
Gastrointestinal surgery	26 (74.3)	27 (77.1)	
Gynecological surgery	4 (11.4)	6 (17.1)	
Urological surgery	5 (14.3)	2 (5.7)	
Duration of anesthesia (mins)	326.29 (96.80)	319.89 (132.41)	0.818
Duration of mechanical ventilation (hours)	16.00 [5.75, 18.50]	14.00 [9.50, 20.50]	0.445

ASA, American Society of Anesthesiologists score, where 1 is a normal healthy patient, 2 is a patient with mild systemic disease, 3 is a patient with severe systemic disease, 4 is a patient with severe systemic disease that is a constant threat to life, and 5 is a moribund patient who is not expected to survive; C-group, control group; CPRM-group, combined physiological recruitment maneuver group; BMI, body mass index; ICU, intensive care unit. All data presented as mean (standard deviation) or median [interquartile range] for continuous variable and number (percentage) for categorical variable

### LUS scores

**Fig. 3:**
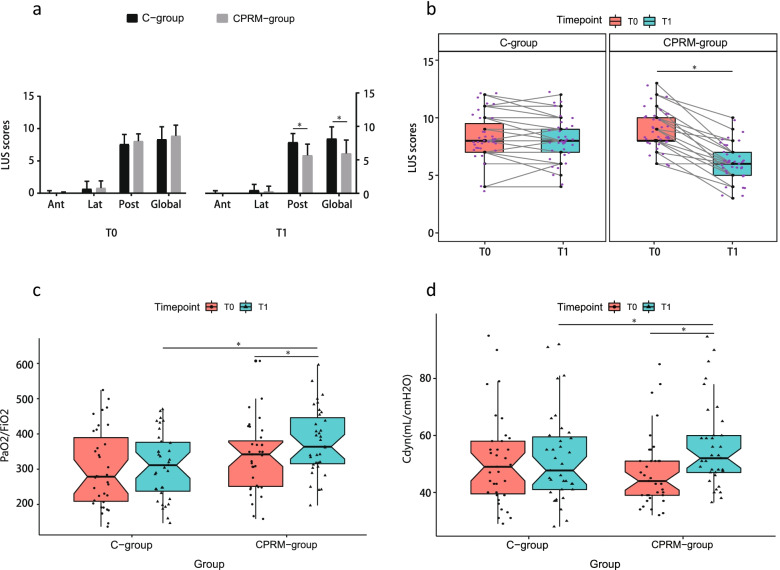
**a** Changes of regional and global LUS scores induced by sigh in lateral position. **b** Individual change of LUS scores induced by sigh in lateral position. **c** Effects of sigh in lateral position on gas exchange. **d** Effects of sigh in lateral position on lung compliance. T0, before recruitment; T1, after recruitment; C-group, control group; CPRM-group, combined physiological recruitment maneuver group; Ant, anterior lung zones; Lat, lateral lung zones; Post, posterior lung zones; LUS, lung ultrasound; PaO2/FiO2, partial pressure of arterial oxygen and fraction of inspiratory oxygen ratio; Cdyn, dynamic compliance; * *p* < 0.05

All ultrasound examinations were successfully performed on every patient and a total of 1680 cine loops were acquired. Before recruitment (time point T0), the highest LUS scores were in the posterior lung zones in C-group and CPRM-group (8.00 [7.00,8.00] vs. 8.00 [7.50,8.00], *p* = 0.24). After recruitment (time point T1), the LUS scores of the posterior lung zones improved the most in CPRM-group, whereas there was no significant change in C-group (6.00 [5.00,6.00] vs. 8.00 [7.00,8.00], *p* = 1.726e-10 < 0.05). There was no significant difference in global LUS scores between C-group and CPRM-group before recruitment (time point T0) (8.00 [7.00, 9.50] vs. 8.00 [8.00, 10.00], *p* = 0.168). After recruitment (time point T1), global LUS scores decreased significantly in CPRM-group but remained unchanged in C-group (6.00 [5.00, 7.00] vs. 8.00 [7.00, 9.00], *p* = 4.463e-11 < 0.05) (Fig. 3a).

Individual change of LUS scores also indicated that sigh in lateral position could effectively improve lung aeration (6.00 [5.00, 7.00] vs. 8.00 [7.00, 9.00], *p* = 4.463e-11 < 0.05) (Fig. 3b). Representative ultrasonographic images at the different time points of one sample patient from each group are shown in Supplementary Figure S1.

### Gas exchange and lung compliance

It is generally accepted that an effective recruitment maneuver should benefit gas exchange and lung compliance.

Oxygenation index, calculated as PaO_2_ divided by FiO_2_, is considered as an useful index of gas exchange. After recruitment (time point T1), the PaO_2_/FiO_2_ improved significantly in CPRM-group (334.42 (96.62) vs. 377.92 (93.73), *p* = 0.013 < 0.05) but remained unchanged in C-group (301.43 (109.79) vs. 309.19 (92.98), *p* = 0.691). There was significant difference in PaO_2_/FiO_2_ between C-group and CPRM-group after recruitment (time point T1) (309.19 (92.98) vs. 377.92 (93.73), *p* = 0.008 < 0.05) (Fig. 3c).

Compliance, the ratio between the change in volume and the change in pressure of the thorax, is a reflection of atelectasis and the effect of recruitment maneuvers. After recruitment (time point T1), the Cdyn improved significantly in CPRM-group (44.00 [39.00, 51.00] vs. 52.00 [47.00, 60.00], *p* = 0.000 < 0.05) but remained unchanged in C-group (49.00 [39.50, 58.00] vs. 47.70 [41.00, 59.50], *p* = 0.062). There was significant difference in Cdyn between C-group and CPRM-group after recruitment (time point T1) (47.70 [41.00, 59.50] vs. 52.00 [47.00, 60.00], *p* = 6.325e-07 < 0.05) (Fig. 3d).

### Complications

No complications associated with sigh in lateral position were observed during the study. There was no hemodynamic instability, carbon dioxide retention and severe acidosis developed in any patient of the two groups (Table 2). Specifically, there was no detectable barotrauma, such as pneumothorax, pneumomediastinum and subcutaneous emphysema. No position-related complications, such as line removal or accidental extubation, were encountered.

**Table 2:** The hemodynamics and blood gas analysis

	C-group *n* = 35	*p* value^※^	CPRM-group *n* = 35	*p* value^#^	*p* value^△^
Before recruitment					
Heart rate (bpm)	71.71 (13.86)		73.71 (14.54)		0.558
Mean artery pressure (mmHg)	94.69 (8.85)		93.77 (12.77)		0.729
CVP (cmH2O)	9.17 (2.15)		8.34 (2.14)		0.111
PH	7.40 (0.06)		7.41 (0.05)		0.153
PaO2 (mmHg)	147.56 (56.16)		167.49 (49.55)		0.120
PaCO2 (mmHg)	36.64 (5.93)		33.34 (5.60)		0.020
After recruitment					
Heart rate (bpm)	71.14 (12.22)	0.701	73.89 (14.96)	0.880	0.528
Mean blood pressure (mmHg)	90.37 (8.88)	0.005	90.97 (10.74)	0.152	0.604
CVP (cmH2O)	8.89 (1.81)	0.549	9.14 (2.07)	0.079	0.456
PH	7.39 (0.04)	0.725	7.41 (0.04)	0.700	0.119
PaO2 (mmHg)	137.11 (41.19)	0.276	151.17 (37.49)	0.0381	0.382
PaCO2 (mmHg)	36.49 (3.74)	0.871	33.70 (4.17)	0.643	0.065

^**※**^ Before recruitment vs. After recruitment in C-group. ^**#**^ Before recruitment vs. After recruitment in CPRM-group. Within-group differences according to Paired t-test for repeated measurements with *p* values less than 0.05 considered significant

^△^ C-group vs. CPRM-group. Between-group differences according to Student’s t-test or ANCOVA for independent samples and with *p* values less than 0.05 considered significant

C-group, control group; CPRM-group, combined physical recruitment maneuver group; CVP, central venous pressure; PaO2, arterial pressure of oxygen; PaCO2, arterial pressure of carbon dioxide

All data presented as mean (standard deviation) or median [interquartile range] for continuous variable and number (percentage) for categorical variable

## Discussion

In this prospective randomized controlled trial, all patients presented varying degrees of aeration modification or atelectasis under general anesthesia lasting for 2 h or longer. Atelectasis is usually associated with decreased lung compliance, impaired oxygenation, increased pulmonary vascular resistance, and lung injury. Therefore, improvement of oxygenation, lung compliance and aeration also correlates well with reopening of atelectatic alveoli. Our study showed that sigh in lateral position could improve gas exchange, lung compliance and aeration with no severe complications. However, there is no difference in the duration of mechanical ventilation between groups.

### Mechanisms contributing to postoperative atelectasis

If general anesthesia does induce atelectasis, then what happens when a patient with normal lungs is placed in a supine position, is anesthetized with anesthetics and neuromuscular blockers, is intubated and ventilated with high concentration oxygen and fixed tidal volume? Three of the most agreed-on mechanisms are dyskinesis, prolonged exposure to high-concentrated oxygen, and ablation of sigh breaths [34]. The neuromuscular blockade could induce paralysis of respiratory muscles and cephalad shift of diaphragm in recumbent human subjects [35], which allows subsequent compression of lower lobe exerted by lungs’ own weight and abdominal pressure [6, 36]. Compression atelectasis has been demonstrated in both anesthetized children and adults [6, 37, 38]. During the induction of general anesthesia, high fraction oxygen could be rapidly absorbed, leading to absorption atelectasis. A study showed that atelectasis was significant in patients receiving 100% oxygen, but it was not obvious and virtually absent in patients receiving 80% and 60% oxygen, respectively [39]. Sigh, a deep inspiration characterized by large volumetric fluctuations, could promote the release and distribution of surfactant on the alveolar surface and distal airways [38, 40], decrease ventilation heterogeneity [41], and improve lung elastance [42]. During general anesthesia, muscle paralysis and fixed tidal volume ventilation would result in an interruption of sigh breath, thus leading to surfactant depletion, alveolar instability, and loss-of-surfactant atelectasis.

### Postoperative atelectasis is a position and pressure-dependent phenomenon

Atelectasis prevails in the most dependent lung area of patients with healthy lungs under general anesthesia [43]. It indicates that atelectasis is a position and pressure-dependent phenomenon (Supplementary Figure S2a). In anesthetized, paralyzed, mechanically ventilated patients with a supine position, the pleural pressure increases gradually along the gravitational axis due to the lung’s own weight [38], the compression through paralyzed diaphragm by abdominal organs could also raise the pleural pressure [6, 36]. While the airway pressure provided by the ventilator is distributed evenly within all pulmonary alveoli from the ventral area to the dorsal area. As a result, the trans-pulmonary pressure, which equals airway pressure minus pleural pressure, decreases gradually from the ventral region to the dorsal region. Each acinus has a critical closing pressure, the minimum trans-pulmonary pressure below which the acinus begins to collapse. Atelectasis would occur when the trans-pulmonary pressure is no longer sufficient to counterbalance the critical closing pressure on the most dorsal dependent parts of the lungs, where the trans-pulmonary pressure is naturally the lowest [44]. While keeping the lungs aerated in the most ventral non-dependent zones, where the trans-pulmonary pressure is generally the highest [17]. No matter in which position the patient is placed, the gravity-dependent lung zones will always be susceptible to airway closure and atelectasis [6, 36, 38].

### The rationality and reliability of sigh in lateral position

Aimed at reopening the collapsed lung, recruitment maneuvers generally refer to ventilator strategies by temporarily increasing airway pressure for several breaths in mechanically ventilated patients [17, 45]. However, studies showed that nearly 50% of patients did not get a significant improvement of gas exchange after 30–120 s of mechanical ventilation with continuous positive airway pressure of 40–50 cm H2O [12, 13]. One situation that limits the efficacy of recruitment maneuvers is that atelectasis is primarily located in the dorsal dependent lung area [46], while the positive airway pressure exerted by recruitment maneuvers would preferentially distribute to the ventral compliant lung zones rather than to the dorsal collapsed alveoli that we intend to reopen.

Sigh in lateral position, a combined physiological recruitment maneuver introduced in our paper, makes full use of the influence of gravity and lung surfactant on respiratory physiology and presents as an effective and low-risk intervention for postoperative atelectasis (Supplementary Figure S2b).

First, this combined physiological recruitment maneuver takes advantage of the relationship between body position and transpulmonary pressure to reopen the collapsed alveoli actively. Like lung collapse, lung recruitment is also a pressure and position-dependent event. An individual alveolus has a minimum opening/closing pressure or a critical transpulmonary pressure at which this particular alveolus would change its state from collapsed to open. In the supine position, lung recruitment is continuous and follows the transpulmonary pressure gradient, starting from the ventral non-dependent zone of the lungs and ending with the dorsal dependent zone, which means that, from the ventral area to the dorsal area, the potential of recruitment maneuver decreases gradually. Hence, lung recruitment could be achieved by changing body position without having to increase airway pressure [17, 45]. Positioning methods also have beneficial effects on oxygenation and prevention of VAP [47–49]. Current studies on postural recruitment are focused primarily on prone positions. Ventilation in prone position could reverse the physiological dorsoventral gradient of the lung [50, 51], could generate high levels of transpulmonary pressure sufficient to exceed minimum airway opening pressure and preferentially reopen the collapsed lung tissue in dorsal dependent zones [52]. However, turning a mechanically ventilated patient to a prone position is challenging, because it needs an easily gathered, closely coordinated, and labor-intensive nursing team [53]. In the same way, sigh in lateral position, which is much easier to be carried out, could also change the physiological dorsal–ventral gradient of the lung and could also favorably inflate the collapsed dorsal dependent lung area [54]. When a patient is turned from supine position to lateral position, previously collapsed lung in the gravity-dependent area is now transferred to a non-gravity-dependent location where the pulmonary alveoli are exposed to lung-distending force of much higher trans-pulmonary pressure. The recruiting effect of lateral position may be owing to the rise of trans-pulmonary pressure caused by a gravity-dependent increase in negative pleural pressure in the upper lung rather than by the elevation of airway pressure as in conventional recruitment maneuvers. Due to the oval shape of the thorax, the left to right diameter is much bigger than the front to back one, when a patient is turned from supine position to lateral position, the trans-pulmonary pressure of the uppermost lung will increase even more dramatically, reaching the minimum opening pressure of the collapsed alveoli in this region, then the lung will reopen actively.

Second, the stabilizing effect of sigh keeps pulmonary alveoli open. Atelectasis would reoccur within 5 min after recruitment maneuver in patients using 100% oxygen [55] or quickly after discontinuation of PEEP in patients receiving 40% oxygen [6, 56], indicating that the collapsed alveoli remain unstable despite having been recruited. Once atelectasis occurs, it is likely to attenuate the effect of pulmonary surfactant so that the newly reopened lung units recollapse again rapidly. Characterized by deep inspiration, the sigh is a physiological homeostatic reflex. Awake adults sigh on average 9 to 10 times per hour unconsciously [57]. For decades, researchers have found that sigh plays a critical role in maintaining pulmonary compliance [58] and minimizing the alveolar-arterial oxygenation difference. One study reported that prolonged mechanical ventilation with adequate but static tidal volume in patients under general anesthesia would result in progressive atelectasis and increased shunting when sighs were absent [57]. It also showed that lung compliance fell 15% and PaO_2_ fell 22% on average without sighs, while after a few minutes of deep, slow, sustained sighs, PaO_2_ rose about 150 mm Hg, indicating that sigh could reduce the intrapulmonary shunting caused by unvarying tidal volume [57]. In this study, we found that sigh could help previously well-ventilated non-dependent lung areas and newly reaerated dependent lung areas keep open even when they become dependent lung regions again after turning the patient from lateral position to its original supine position. Likely due to sigh breath could increase surfactant release exponentially with stretching in alveolar type II cells, promote the spreading and biological activity of surfactant on the surface of the alveolar [38, 40].

Sigh in lateral position was also well-tolerated hemodynamically and was not stopped in any of the patients. No other serious complications, such as pneumothorax or barotrauma, occurred during the recruitment procedure.

### LUS is a useful tool for monitoring lung aeration changes

Thanks to its reliability, feasibility and portability, point-of-care ultrasound has been extensively developed in the field of perioperative monitoring and critical care for decades. Recently, the quick development of LUS has provided a new bedside tool for the timely diagnosis and rapid assessment in critical patients. LUS also has great value for bedside assessment of lung aeration, especially, allows tracking of perioperative atelectasis and monitoring the recruiting effect of recruitment maneuver in critically ill patients [29–31, 33, 59].

Our study showed that LUS is a non-invasive and sensitive tool for real-time imaging of lung aeration. LUS has a gradation of scores, as normal aeration, moderate, severe, and complete loss of aeration, according to the presence of A-lines, B-lines and consolidation, therefore LUS is not dedicated to consolidation assessment only [60]. LUS could reflect any degree of aeration modification sensitively, even if lung consolidation is not involved.

### Limitations

There are some limitations in our study.

One shortcoming is the lack of baseline ultrasound images of patients before the operation. However, preoperative CT scans have been performed to exclude patients with pulmonary lesions and abnormal imaging. Therefore, we can assume that all patients included having the same baseline ultrasound images.

Our study lacks data on the inter-operator variability in the assessment of lung aeration loss. In this study, LUS was performed by the same advanced expert on point of care ultrasound, besides, LUS relies on the recognition of basic and simple ultrasound pattern and previous studies have already demonstrated a high inter-observer agreement and low variation of this technique [61, 62].

Dynamic rather than static lung compliance was applied in this study since it is a technical challenge to execute inspiratory hold and measure plateau pressure on the ventilator. However, because subjects with chronic obstructive lung disease (COPD) have been excluded from our study, the peak pressure of included patients does not vary much from the plateau pressure and thus Cdyn would not differ too much from Cstat.

Sigh in lateral position might lack relevance in clinical practice. We can’t “keep” patients 2 h in mechanical ventilation with sigh in lateral position in PACU.

Statistical differences in aeration and mechanics exist and are related to sigh in lateral position, clinical relevance are arguable. Large-scale clinical studies should be performed to verify it in the future.

Finally, our research is a single-center study focusing only on short-term effects in adults only, such as LUS scores, Cdyn and PaO_2_/FiO_2_. Future large-scale research should also focus on long-term effects in adults and children, such as ventilator-associated pneumonia and duration of hospitalization.

## Conclusion

As assessed by LUS, sigh in lateral position could actively reopen atelectasis without elevating airway pressure and causing severe side effects of traditional recruitment maneuver. Further large-scale studies are necessary to evaluate it’s long-term effects on pulmonary complications and hospital length of stay.

## Supplementary Information

**Additional file 1:** **Table S1**. LUS grading system.

**Additional file 2:** **Fig S1**. Lung aeration changes in the right posteroinferior quadrant of one patient from each group at different time point. After open abdominal surgery under general anesthesia lasting for 2 hours or longer (T0), the preoperative normal lung underwent lung aeration loss resulting in the appearance of multiple coalescent B lines in both groups. After recruitment (T1), sigh in lateral position (CPRM-group) have led to re-aeration (coalescent B-lines changing into A-lines) in the sample patient. However, lung aeration loss (coalescent B-lines transforming into subpleural tissue-like pattern) was seen in the sample patient from C-group. T0, before recruitment; T1, after recruitment; C-group, control group; CPRM-group, combined physiological recruitment maneuver group; Asterisks denote B lines; White arrowheads denote consolidation; White arrows denote A-lines.

**Additional file 3:** **Fig S2**. (a) Postoperative atelectasis is a position and pressure-dependent phenomenon. In anesthetized patients with supine position, the negative pleural pressure increases gradually along the gravity vector because of the lungs’ own weight and the compression of abdominal organs, while the positive airway pressure delivered by the ventilator is distributed homogeneously within the lungs. Therefore, the trans-pulmonary pressure (airway pressure minus pleural pressure) and the tendency of lung collapse decrease gradually from the dorsal dependent zones to the ventral non-dependent areas. The opposite is also true when it comes to opening pressure and potential of lung recruitment. (b) Schematic diagram of sigh in lateral position. The maneuver consists of periodic sigh breaths and sequential changes in position from the supine position to the lateral position and then back to the supine position again. Due to the oval shape of the chest, the gradient of trans-pulmonary pressures is much greater in the lateral position than in the supine position. Thus, the upper half of the lungs is re-aerated easily in the lateral position. Once re-aerated, the upper half can maintain its “open lung” condition even when the patient is turned to the opposite side provided sighs are applied. Finally, both lungs remain “open” although the patient has returned to the previous supine position. Ptp, trans-pulmonary pressure.

## Data Availability

The dataset(s) supporting the conclusions of this article is (are) included within the article (and its additional file(s)).

## Abbreviations

LUS: Lung ultrasound
CPRM: Combined physiological recruitment maneuver
C-group: Control group
CPRM-group: Combined physiological recruitment maneuver group
Cdyn: Dynamic compliance
PaO_2_: Partial pressure of arterial oxygen
FiO_2_: Fraction of inspiratory oxygen
PaO_2_/FiO_2_: Partial pressure of arterial oxygen and fraction of inspiratory oxygen ratio
CT: Computed tomography
MRI: Magnetic resonance imaging
EIT: Electric impedance tomography
RCT: Randomized controlled trial
Vt: Tidal volume
RR: Respiratory rate
I/E: Inspiratory-to-expiratory ratio
PEEP: Positive end-expiratory pressure
PaCO_2_: Partial pressure of arterial carbon dioxide
Ppeak: Peak airway pressure
Pplat: Plateau inspiratory pressure
OI: Oxygenation index; HR, heart rate
MAP: Mean arterial pressure
CVP: Central venous pressure
SD: Standard deviation
IQR: Interquartile range
ANCOVA: Analysis of covariance
Cstat: Static compliance
COPD: Chronic obstructive lung disease
GA: General anesthesia
ASA: American Society Anesthesiologists score
FRC: Functional Residual Capacity
VAP: Ventilator Acquired Pneumonia
US-RAS: Ultrasound re-aeration score
DNA: Deoxyribonucleic acid
ARDS: Acute respiratory distress syndrome
ALI: Acute lung injury
MB: Multi-beam
THI: Tissue harmonic imaging
RM: Recruitment maneuver
CI: Confidence interval
RASS: Richmond Agitation-Sedation Scale
Ptp: Transpulmonary pressure
