# Supplementary material for: Effect of sigh in lateral position on postoperative atelectasis in adults assessed by lung ultrasound: a randomized, controlled trial
Source: BMC Anesthesiol. 2022 Jul 11;22:215. doi: 10.1186/s12871-022-01748-9 (PMC9275275; doi:10.1186/s12871-022-01748-9)
Supplement: Supplementary file 1 — Additional file 1: Table S1. LUS grading system. [file 12871_2022_1748_MOESM1_ESM.docx]

Table S1. LUS grading system

|  | Normal Aeration | Mild Loss of Aeration | Moderate Loss of Aeration | Severe Loss of Aeration |
| --- | --- | --- | --- | --- |
| LUS patterns | N | B1 | B2 | C |
| LUS scores | 0 | 1 | 2 | 3 |
| LUS manifestations | 0-2 B lines or A-lines | ≥3 well-defined B-lines | Multiple coalescent B-lines | Subpleural tissue-like pattern and air bronchogram in consolidation |
| Representative LUS images | 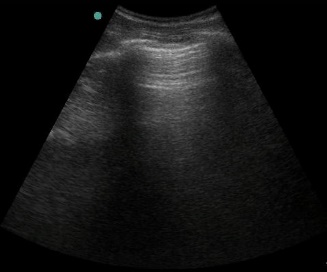 | 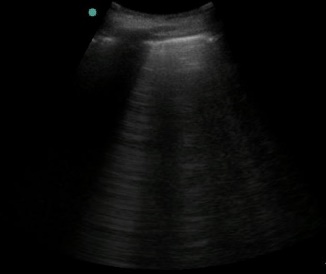 | 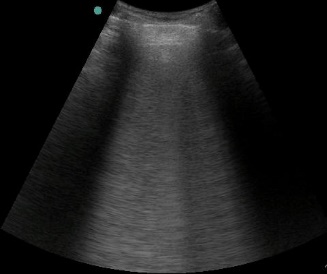 | 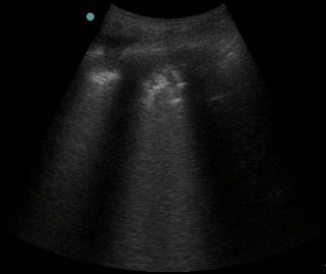 |

LUS, lung ultrasound.
