# Supplementary material for: Effect of sigh in lateral position on postoperative atelectasis in adults assessed by lung ultrasound: a randomized, controlled trial
Source: BMC Anesthesiol. 2022 Jul 11;22:215. doi: 10.1186/s12871-022-01748-9 (PMC9275275; doi:10.1186/s12871-022-01748-9)
Supplement: Supplementary file 2 — Additional file 2: Fig S1. Lung aeration changes in the right posteroinferior quadrant of one patient from each group at different time point. After open abdominal surgery under general anesthesia lasting for 2 hours or longer (T0), the preoperative normal lung underwent lung aeration loss resulting in the appearance of multiple coalescent B lines in both groups. After recruitment (T1), sigh in lateral position (CPRM-group) have led to re-aeration (coalescent B-lines changing into A-lines) in the sample patient. However, lung aeration loss (coalescent B-lines transforming into subpleural tissue-like pattern) was seen in the sample patient from C-group. T0, before recruitment; T1, after recruitment; C-group, control group; CPRM-group, combined physiological recruitment maneuver group; Asterisks denote B lines; White arrowheads denote consolidation; White arrows denote A-lines. [file 12871_2022_1748_MOESM2_ESM.pdf]

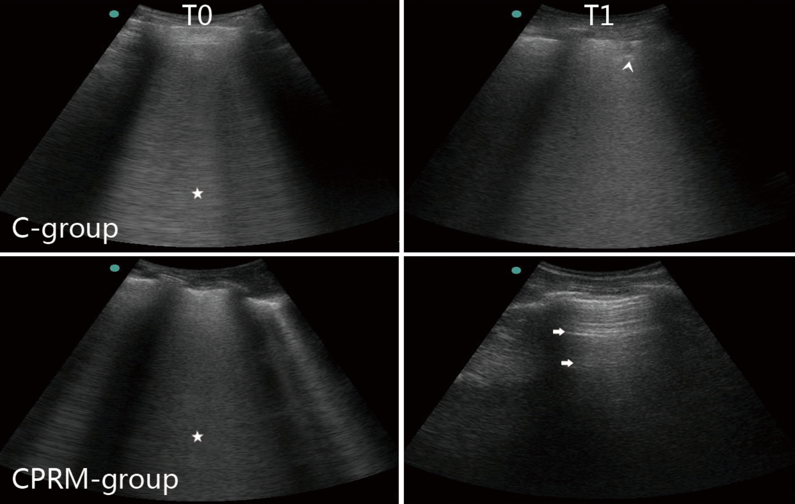
T0
T1
C-group
CPRM-group

Fig S1. Lung aeration changes in the right posteroinferior quadrant of one patient from each group at different time point.

After open abdominal surgery under general anesthesia lasting for 2 hours or longer (T0), the preoperative normal lung underwent lung aeration loss resulting in the appearance of multiple coalescent B lines in both groups. After recruitment (T1), sigh in lateral position (CPRM-group) have led to re-aeration (coalescent B-lines changing into A-lines) in the sample patient. However, lung aeration loss (coalescent B-lines transforming into subpleural tissue-like pattern) was seen in the sample patient from C-group.

T0, before recruitment; T1, after recruitment; C-group, control group; CPRM-group, combined physiological recruitment maneuver group; Asterisks denote B lines; White arrowheads denote consolidation; White arrows denote A-lines.
