# Supplementary material for: Effect of sigh in lateral position on postoperative atelectasis in adults assessed by lung ultrasound: a randomized, controlled trial
Source: BMC Anesthesiol. 2022 Jul 11;22:215. doi: 10.1186/s12871-022-01748-9 (PMC9275275; doi:10.1186/s12871-022-01748-9)
Supplement: Supplementary file 3 — Additional file 3: Fig S2. (a) Postoperative atelectasis is a position and pressure-dependent phenomenon. In anesthetized patients with supine position, the negative pleural pressure increases gradually along the gravity vector because of the lungs’ own weight and the compression of abdominal organs, while the positive airway pressure delivered by the ventilator is distributed homogeneously within the lungs. Therefore, the trans-pulmonary pressure (airway pressure minus pleural pressure) and the tendency of lung collapse decrease gradually from the dorsal dependent zones to the ventral non-dependent areas. The opposite is also true when it comes to opening pressure and potential of lung recruitment. (b) Schematic diagram of sigh in lateral position. The maneuver consists of periodic sigh breaths and sequential changes in position from the supine position to the lateral position and then back to the supine position again. Due to the oval shape of the chest, the gradient of trans-pulmonary pressures is much greater in the lateral position than in the supine position. Thus, the upper half of the lungs is re-aerated easily in the lateral position. Once re-aerated, the upper half can maintain its “open lung” condition even when the patient is turned to the opposite side provided sighs are applied. Finally, both lungs remain “open” although the patient has returned to the previous supine position. Ptp, trans-pulmonary pressure. [file 12871_2022_1748_MOESM3_ESM.pdf]

a

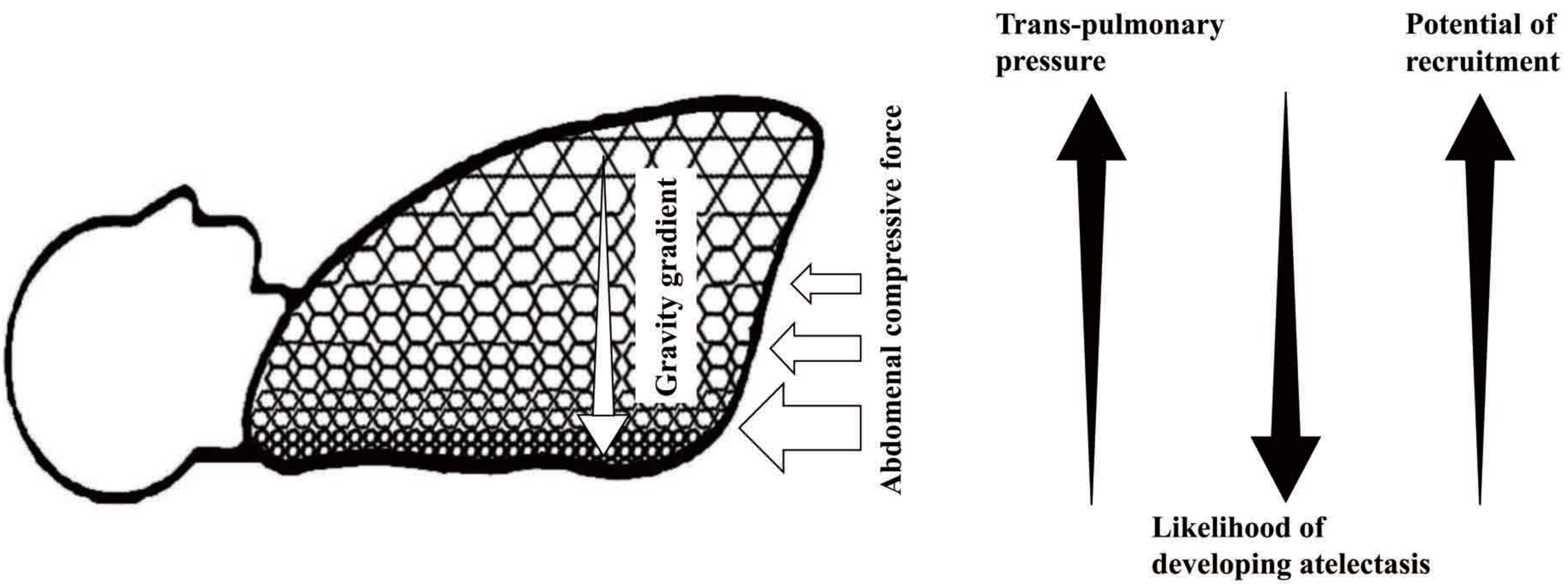

Trans-pulmonary pressure
Potential of recruitment
Gravity gradient
Abdomenal compressive force
Likelihood of developing atelectasis

b

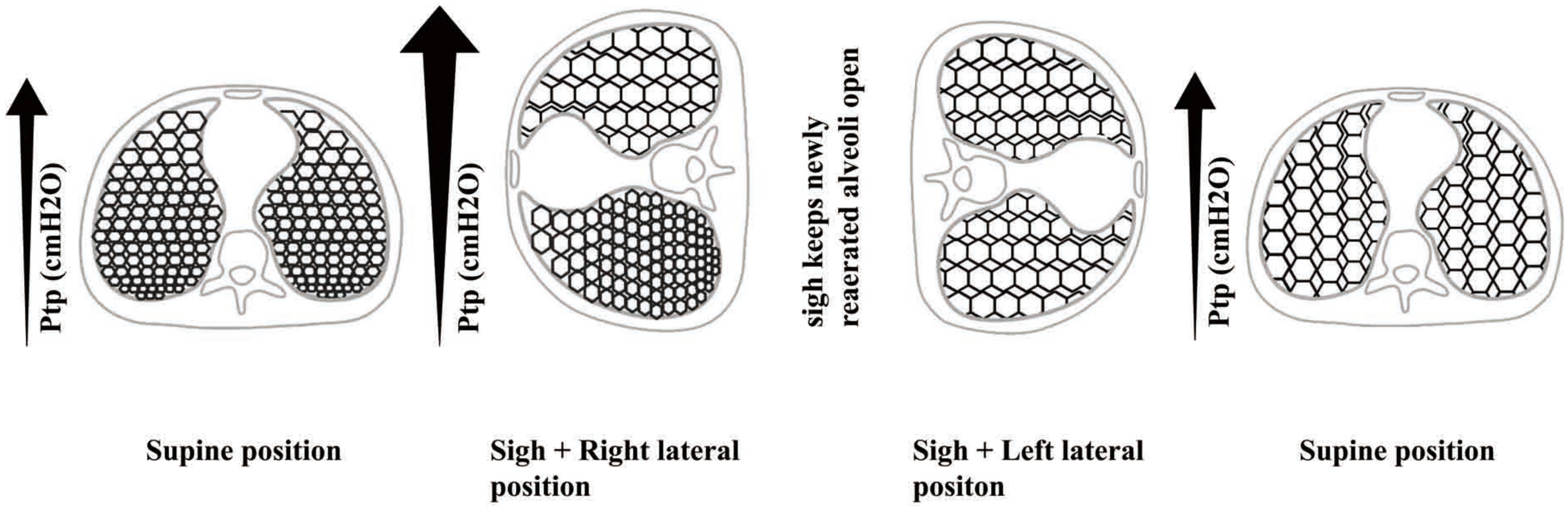

Ptp (cmH2O)
Ptp (cmH2O)
sigh keeps newly reaerated alveoli open
Ptp (cmH2O)
Supine position
Sigh + Right lateral position
Sigh + Left lateral positon
Supine position

Fig S2. (a) Postoperative atelectasis is a position and pressure-dependent phenomenon.

In anesthetized patients with supine position, the negative pleural pressure increases gradually along the gravity vector because of the lungs' own weight and the compression of abdominal organs, while the positive airway pressure delivered by the ventilator is distributed homogeneously within the lungs. Therefore, the trans-pulmonary pressure (airway pressure minus pleural pressure) and the tendency of lung collapse decrease gradually from the dorsal dependent zones to the ventral non-dependent areas. The opposite is also true when it comes to opening pressure and potential of lung recruitment.

(b) Schematic diagram of sigh in lateral position. The maneuver consists of periodic sigh breaths and sequential changes in position from the supine position to the lateral position and then back to the supine position again. Due to the oval shape of the chest, the gradient of trans-pulmonary pressures is much greater in the lateral position than in the supine position. Thus, the upper half of the lungs is re-aerated easily in the lateral position. Once re-aerated, the upper half can maintain its "open lung" condition even when the patient is turned to the opposite side provided sighs are applied. Finally, both lungs remain "open" although the patient has returned to the previous supine position.

Ptp, trans-pulmonary pressure.
